# Reproducibility and normalization of reactive hyperemia using laser speckle contrast imaging

**DOI:** 10.1371/journal.pone.0244795

**Published:** 2021-01-07

**Authors:** Behnia Rezazadeh Shirazi, Rudy J. Valentine, James A. Lang

**Affiliations:** Department of Kinesiology, Iowa State University, Ames, Iowa, United States of America; University of Massachusetts Boston, UNITED STATES

## Abstract

**Background:**

Impaired perfusion indices signal potential microvascular dysfunction preceding atherosclerosis and other cardiometabolic pathologies. Post-occlusive reactive hyperemia (PORH), a vasodilatory response following a mechanically induced ischemia, is a transient increase in perfusion and can assess microvascular function. The greatest blood flow change corresponding to the first minute of hyperemia (represented by time-to-peak, hyperemic velocity, AUC within 1^st^ min) has been shown to indicate microvascular dysfunction. However, the reproducibility of these temporal kinetic indices of the PORH response is unknown. Our aim was to examine the inter- and intra-day reproducibility and standardization of reactive hyperemia, with emphasis on the kinetic indices of PORH, using laser speckle contrast imaging (LSCI) technique.

**Methods and results:**

Seventeen healthy adults (age = 24 ± 3 years) completed three PORH bouts over two lab visits. LSCI region of interest was a standardized 10 cm region on the dominant ventral forearm. A 5-min brachial artery occlusion period induced by inflating an arm cuff to 200 mmHg, preceded a 4-min hyperemic period. Inter- and intra-day reliability and reproducibility of cutaneous vascular conductance (LSCI flux / mean arterial pressure) were determined using intraclass correlation (ICC) and coefficient of variation (CV%). Maximal flow and area under the curve standardized to zero perfusion showed intra- and inter-day reliability (ICC > 0.70). Time to maximal flow (TMF) was not reproducible (inter-day CV = 18%). However, alternative kinetic indices such as 1-min AUC and overshoot rate-of-change (ORC), represented as a piecewise function (at 5s, 10s, 15s, and 20s into hyperemia), were reproducible (CV< 11%). Biological zero was a reliable normalization point.

**Conclusion:**

PORH measured with LSCI is a reliable assessment of microvascular function. However, TMF or its derived hyperemic velocity are not recommended for longitudinal assessment. Piecewise ORC and 1-min AUC are reliable alternatives to assess the kinetic response of PORH.

## 1. Introduction

Laser-based optical techniques are routinely used to measure cutaneous microvascular function in response to various perturbations. The most common tests are reactive hyperemia, local thermal hyperemia, and hyperemia following administration of acetylcholine or sodium nitroprusside. Each of these tests invokes a relatively different mechanism of microvascular function and vary in reproducibility and ease of implementation [1,2]. Cutaneous post-occlusive reactive hyperemia (PORH), characterized by markedly increased blood flow supplying a distal extremity following a period of arterial occlusion (typically 3–5 min), is predominantly mediated by the involvement of sensory nerve axonal reflex and endothelium-derived hyperpolarizing factors (EDHF). It has been demonstrated that nitric oxide (NO) does not influence reactive hyperemia at the skin microcirculatory level [3–6]. Moreover, the direct role of prostaglandins on skin reactive hyperemia is unclear. Although there are reports to the contrary [4] it appears that skin PORH is not mediated by prostaglandins [3,7,8]. However, it has been shown that cyclooxygenase inhibition unmasks the NO role in the cutaneous PORH response suggesting a feedback modulation between NO and prostaglandin biosynthetic pathways involved in the PORH response [9].

Considering the mechanisms involved, PORH may be an effective tool to assess microvascular function and cardiovascular disease (CVD) progression. Previous studies have indicated an association between an impaired PORH response and several disease states including, chronic kidney disease [10], coronary artery disease [11], diabetes [12,13], and peripheral arterial disease [14]. Furthermore, statin [4] or antihypertensive therapies [15] improve the PORH response in patients with microvascular dysfunction. Although PORH may be a useful preclinical indicator of CVD progression, its clinical application requires a reliable longitudinal, assessment. Hence, it is critical for PORH measurements to have good to excellent test-retest or inter-day reliability for assessing microvascular function. The widespread clinical use of PORH has been limited in part due to the laser optical methods used as well as inconsistencies in analysis methods.

Various forearm PORH indices measured with single-point laser doppler flowmetry (LDF) are less reliable (e.g. CV AMP_RF_ = 33% and MF%RF = 32%) compared to the indices measured using laser speckle contrast imaging (LSCI) (e.g. CV AMP_RF_ = 11% and MF%RF = 14%) [16]. A major limitation of LDF is the small field of coverage [17], which increases test-retest data variability due to the inter-site heterogeneous capillary density of the skin microvasculature [18]. In contrast, a full-field microvascular measurement provided by LSCI minimizes the data variability associated with the skin site capillary heterogeneity. However, normalization of data using LSCI is unclear. In addition to normalizing to the invariable zero flux, adjusting flux signal to physiological reference points with inherent flux variability such as resting flow (RF) or biological zero (BZ) have been used.

Previous inter-day reproducibility studies on PORH using LDF [16,17,19] or LSCI [16,17,20] focus primarily on the same set of PORH indices (e.g. MF, AMP_RF_, MF%RF, and AUC normalized to zero flux). However, examining kinetic variables within the first minute of hyperemia, such as time-to-peak or velocity of the PORH response (i.e., both indicators of vascular resistance), have been shown to discriminate microvascular functional responses in clinical populations [13,21]. Comparatively, there is little to no reproducibility data reported on PORH kinetic indices particularly when using LSCI [22].

The purpose of this study was twofold. First, we determined the reliability of a normalized PORH response with different reference levels (e.g. zero flux, RF, and BZ). Second, we evaluated the reproducibility of PORH kinetics as a function of variable (e.g. TMF) and static (e.g. 5-20s ORC) temporal parameters and discussed the possible physiological implications of each method. We hypothesized that normalizing to biological zero (i.e. the flux during occlusion) would remove the additive effects of this flux index while preserving reproducibility of the PORH signal. Furthermore, we hypothesized that PORH indices that are based upon a variable temporal index are less reproducible than when variables are based upon a static temporal index.

## 2. Materials and methods

### Participants

Seventeen healthy young men and women (24 ± 3 years, 13M 4F) participated in the study. All participants were normotensive, non-smokers, not obese, without a history of cardiovascular or skin disease, and not taking medications or supplements that alter vascular function. Prior to an experiment visit, participants refrained from strenuous exercises for 24 h, abstained from caffeine and alcohol for 12 h, and fasted for 3 h. The study was approved by the Iowa State University Institutional Review Board (IRB# 17633) and adhered to the Declaration of Helsinki. All participants gave verbal and written consent prior to their enrollment in the study.

### Study design

The study consisted of two lab visits (V_A_, V_B_) interspaced by 4–14 days except for one individual with visits spaced 30 days apart. Body composition was assessed using bioelectrical impedance analysis (InBody720, Los Angeles, USA) prior to the beginning of data collection on the first visit. A total of 3 PORH responses were measured on the dominant forearm over the span of the study. V_A_ and V_B_ consisted of one and two PORH responses respectively. The order of the visits was *a priori* randomly assigned for each subject.

Upon arriving to the laboratory (Ta = 22 ± 1°C, RH = 41 ± 15%), participants rested in semi-reclined position for 20–30 min. While reclined, the dominant arm was secured with a vacuum cushion to minimize forearm movement. The site for skin blood flow measurement was standardized to a 10 cm region defined 3–13 cm below the antecubital fossa for all participants. Measurements were performed in the dominant arm to ensure consistency within and between subjects. A laser speckle contrast imager (LSCI; Moor FLPI-2, Moor Instruments Ltd, Devon, UK), which measures changes in skin perfusion or red blood cell flux pattern, was placed 15–25 cm above the standardized region. The LSCI recorded flux data at 25 Hz at a time constant of 1.0 s and 785 nm wavelength. Camera to forearm distance and autofocused spatial resolution were replicated for inter-day measurements.

After establishing a steady baseline skin blood flow, a pneumatic blood pressure cuff (D.E. Hokanson, Inc, Bellevue, Washington, USA) was inflated on the dominant arm to 200 mmHg for 5 minutes. Flux recording was resumed 30s before cuff deflation and continuously recorded throughout the hyperemic response. Blood pressures were collected using an automated sphygmomanometer (Dinamap, GE, Medical Systems, Milwaukee, USA) from the contralateral arm during baseline, prior to cuff deflation, and 4 minutes post cuff deflation. In the case of V_B_, 30 minutes of time was given between the two PORH responses (V_B1_ and V_B2_).

### Data collection and analysis

**Fig 1:**
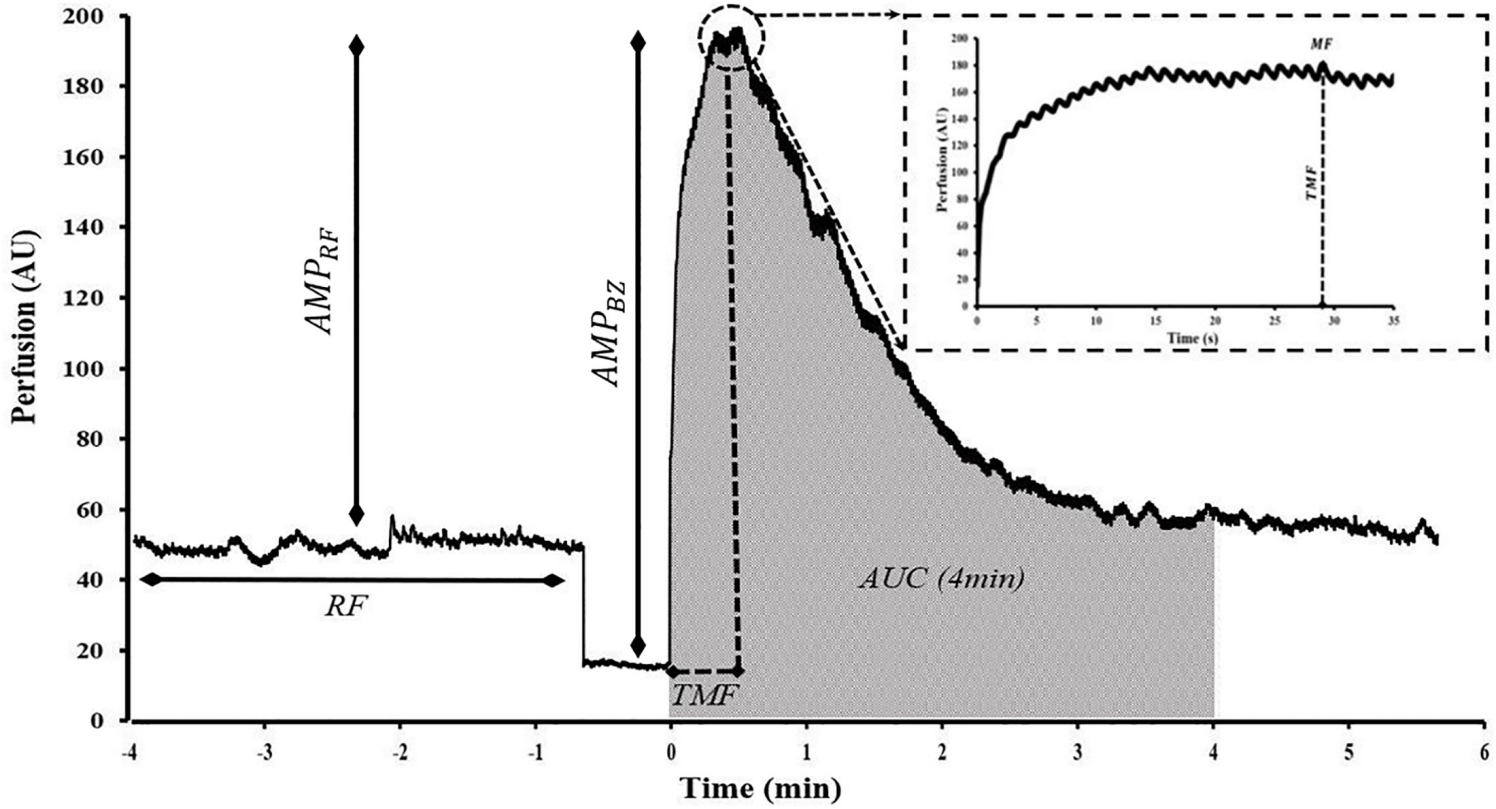
Commonly examined post-occlusive reactive hyperemia parameters shown in a representative PORH response. Resting flow (RF), maximal flow (MF), time to maximal flow (TMF), amplitude of the MF normalized to RF and BZ (AMP_RF_ and AMP_BZ_) and 4-min post cuff-deflation area under hyperemic curve (4-min AUC) normalized to zero perfusion are shown. Time = 0 corresponds to cuff deflation. Magnified tracing on the top right shows the hyperemic response 30s post-cuff deflation.

**Fig 2:**
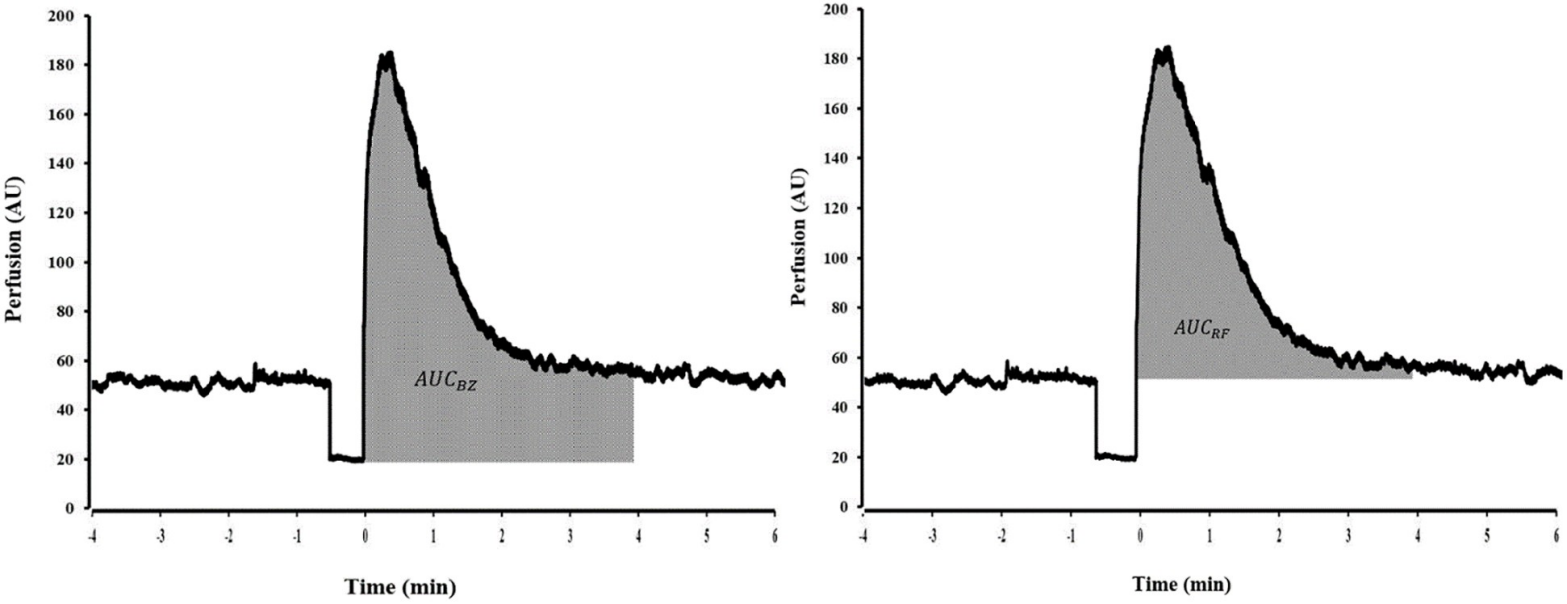
Normalization methods for the area under the curve (AUC). The AUC subtracted from biological zero (AMP_BZ_) or subtracted from resting flow (AMP_RF_).

LSCI flux during PORH was expressed as arbitrary units (AU) and illustrated in Fig 1. *Common PORH indices* were defined as follows: resting Flow (RF) was calculated as the mean of at least 1 min of stable flow prior to occlusion. Maximal Flow (MF) was defined as the highest flux after cuff deflation. Amplitude of the PORH response was expressed as the difference between MF and RF (AMP_RF_) or MF and biological zero (AMP_BZ_). Percentage increase (PI) was the percentage ratio of AMP_RF_ over the RF. Lastly, the 4-min AUC was expressed as a function of RF and biological zero (AUC_RF_ and AUC_BZ_) as shown in Fig 2.

**Fig 3:**
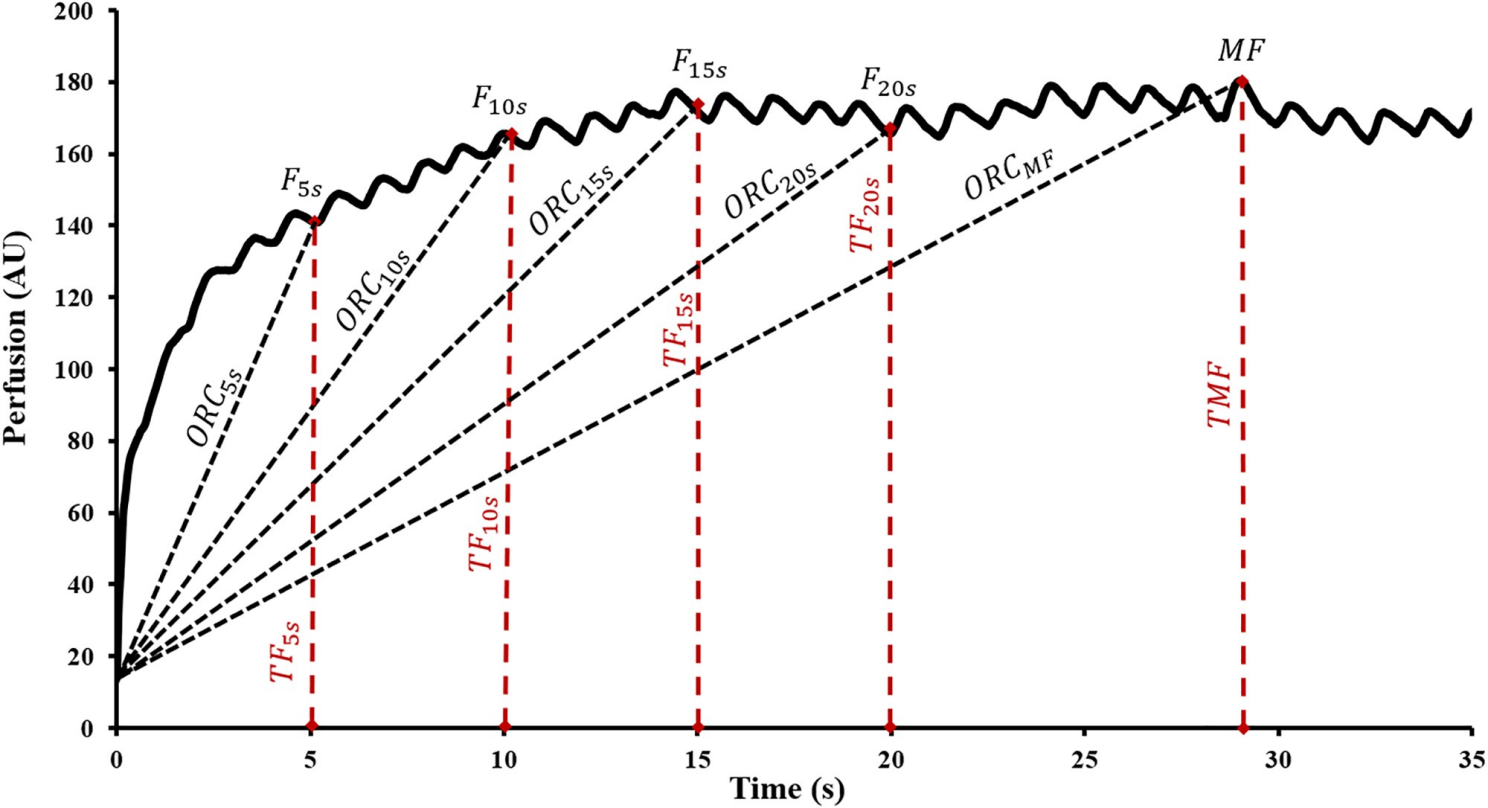
Piecewise overshoot rate of change. 5s intervals from 5-20s (ORC_5s,10s,15s,20s_) hyperemic response illustrated in this tracing obtained from experimental data. Time = 0 corresponds to cuff deflation.

*PORH kinetic indices*, or those variables reflecting the greatest rate of change occurring after cuff release, of the first minute of hyperemia are defined as follows: Time to Maximal Flow (TMF) was the time lapsed from point of deflation to reach MF. A 1-min area under the hyperemic curve (AUC) with respect to zero perfusion was determined. AUC index was calculated as the ratio of 1-min post deflation AUC over 1-min resting flow AUC prior to occlusion. Flux data at 5s, 10s, 15s, and 20s were expressed as functions of biological zero and further divided by the corresponding time durations to elicit the piecewise overshoot rate of change (ORC_5s,10s,15s,20s_) depicted in Fig 3. MF was further expressed from biological zero and divided by TMF to obtain ORC_MF_. All flux data were divided by the mean arterial pressure (diastolic pressure + 1/3 pulse pressure) and reported as cutaneous vascular conductance (CVC). Calculations for several of the parameters are included in Table 1.

**Table 1:** PORH parameters and their mathematical expressions.

PORH Parameters	Definitions
AMP_RF_(AU)	MF − RF
AMP_BZ_(AU)	MF − BZ
MF%RF	$(\frac{\text{MF}-\text{RF}}{\text{RF}})\times 100$
MF%BZ	$(\frac{\text{MF}-\text{BZ}}{\text{BZ}})\times 100$
AUC_RF_(AU · s)	AUC − (240 × RF)
AUC_BZ_(AU · s)	AUC − (240 × BZ)
AUC index	$\frac{1\text{min}\;\text{post}\;\text{deflation}\;\text{AUC}}{1\text{min}\;\text{pre}\;\text{inflation}\;\text{AUC}}$
ORC_MF_(AU · s^-1^)	$\frac{\text{MF}-\text{BZ}}{\text{TMF}}$
ORC_5s,10s,15s,20s_(AU · s^-1^)	$\frac{{\text{F}}_{5\text{s},10\text{s},15\text{s},20\text{s}}-\text{BZ}}{5\text{s},\;10\text{s},\;15\text{s},\;20\text{s}}$

### Statistical analysis

All PORH measures were normally distributed, as assessed by the Shapiro-Wilk test. The inter- and intra-day reliability (V_A_ vs V_B1_, V_B1_ vs V_B2)_ of each PORH measure was determined using intraclass correlation coefficient (ICC), the 95% limits of agreement, and the coefficient of variation (CV). The ICC model was based upon a single measurement, absolute agreement, and 2-way mixed model [23]. ICC values less than 0.5, between 0.5 and 0.75, between 0.75–0.9, and above 0.9 were considered poor, moderate, good, and excellent agreement, respectively [23]. Reproducibility was expressed as within subject CV calculated using the root mean square method [24]. CV values less than 10%, between 10–25%, and more than 25% were defined as good, moderate, and poor reproducibility respectively [25]. A paired T-test was performed between inter-day (V_A_, V_B1_) and intra-day (V_B1_, V_B2_) measurement indices. A p-value < 0.05 was considered statistically significant. An *a priori* sample size analysis indicated that 15 participants were needed to detect a level of agreement (LOA) of 0.6 with a two-observation test-retest design and an α < 0.05 and 80% power. Data are expressed as mean ± SD. Statistical analyses were performed using SPSS (SPSS version 24, IBM, Armonk, NY, USA).

## 3. Results

### Common PORH indices

The mean body mass index was 23 ± 3 kg/m^2^, body fat percentage was 18 ± 8%, systolic and diastolic arterial pressures were 119 ± 10 mmHg and 65 ± 7 mmHg, and mean arterial pressure (MAP) was 82 ± 7 mmHg. The mean values of commonly used PORH parameters along with their inter- and intra-day agreement and reproducibility values are shown in Table 2. MAP had good reproducibility (Inter-day CV = 4% and Intra-day CV = 2%) RF, MF, and 4-min AUC normalized to zero had good reproducibility (CV < 10%) with good reliability values for RF and MF (0.75 < ICC < 0.9). Reproducibility for the TMF was moderate (10% < CV < 25%) while its ICC limits of agreement had a broad spread ranging from poor to good. Overall, AMP_RF_ was more reproducible and reliable than the MF%RF.

**Table 2:** Descriptive and reproducibility statistics for commonly used post-occlusive reactive hyperemia parameters.

	Descriptive Statistics (CVC)			Reproducibility Statistics (CVC)			
	Mean ± SD			INTRADAY (V_B1_ & V_B2_)		INTERDAY (V_A_ & V_B1_)	
	V_A_	V_B1_	V_B2_	ICC (LOA)	CV (%)	ICC (LOA)	CV (%)
RF	0.62 ± 0.17	0.62 ± 0.14	0.60 ± 0.15	0.89 (0.73, 0.96)	6.47	0.89 (0.72, 0.96)	6.72
MF	2.41 ± 0.70	2.32 ± 0.56	2.35 ± 0.73	0.88 (0.70 0.95)	5.13	0.77 (0.48, 0.91)	9.49
TMF	19.3 ± 5.68	19.0 ± 6.82	18.9 ± 5.11	0.65 (0.25, 0.86)	11.71	0.52 (0.06, 0.80)	17.75
4-min AUC	282 ± 88.5	274 ± 70.4	262 ± 67.2	0.93 (0.79, 0.97)	4.88	0.73 (0.39, 0.89)	9.33
MF%RF	300 ± 74.0	282 ± 62.6	285 ± 53.2	0.70 (0.34, 0.88)	10.74	0.51 (0.07, 0.79)	12.65
AMP_RF_	1.81 ± 0.60	1.71 ± 0.46	1.75 ± 0.60	0.85 (0.64, 0.94)	6.65	0.66 (0.28, 0.86)	12.61

Mean ± SD, intraclass correlation (ICC), 95% limits of agreement, and coefficient of variation (CV) for the three PORH responses (V_A_, V_B1_, V_B2_) are shown. PORH indices shown are resting flow (RF), maximal flow (MF), time to maximal flow (TMF), area under 4-min hyperemic curve (4-min AUC) normalized to zero perfusion, max flow as a percentage of resting flow (MF%RF), and amplitude from max flow normalized to resting flow (AMP_RF_). Where applicable, values are represented as cutaneous vascular conductance (CVC).

**Fig 4:**
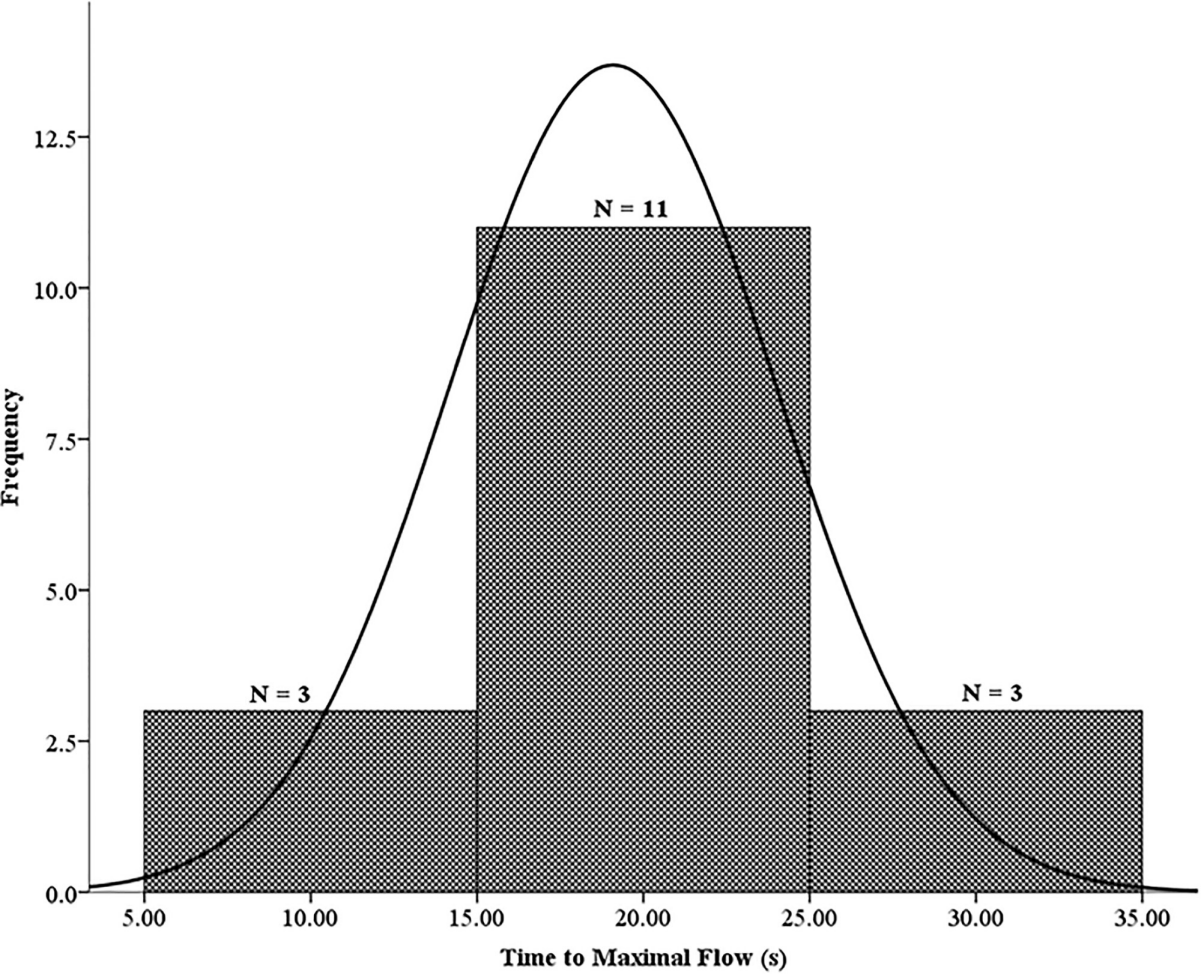
Average TMF (19.1 ± 4.9s) of the three PORH responses (V_A_, V_B1_, V_B2_). These data were normally distributed. The skewness and kurtosis were -0.064 and -0.642, respectively.

Degree of normality for the averaged TMFs of the three post-occlusive reactive hyperemia responses (V_A_, V_B1_, V_B2_) is shown in Fig 4. Out of the 17 participants, 11 expressed an overall averaged TMF of 15-25s while 3 reached maximal flow 5-15s and 25-30s after cuff deflation.

### PORH kinetic indices

Less studied and novel parameter of PORH along with their inter- and intra-day agreement and reproducibility values are shown in Table 3. AMP_BZ_ was more reproducible and reliable than MF%BZ. AUC (4-min) normalized to BZ was more reproducible and more reliable than when it was normalized to the RF. Reproducibility and reliability of the 1-min AUC, similar to that of the 4-min AUC, were highest when normalized to zero perfusion. Although AUC index exhibited good reproducibility, the inter-day reliability was poor (ICC = 0.33). Perfusion ascent rate quantified as overshoot rate of change from BZ calculated at 5s, 10s, 15s, and 20s (ORC_5s,10s,15s,20s_) post-cuff deflation had better reproducibility and reliability than when this rate was calculated at the TMF (ORC_MF_).

**Table 3:** Descriptive and reproducibility statistics for inter- and intra-day novel PORH parameters.

	Descriptive Statistics (CVC)			Reproducibility Statistics (CVC)			
	Mean ± SD			INTRADAY (V_B1_ & V_B2_)		INTERDAY (V_A_ & V_B1_)	
	V_A_	V_B1_	V_B2_	ICC (LOA)	CV (%)	ICC (LOA)	CV (%)
BZ	0.21 ± 0.07	0.23 ± 0.08	0.23 ± 0.08	0.80 (0.53, 0.92)	10.40	0.64 (0.26, 0.85)	14.51
MF%BZ	1072 ± 317	973 ± 244	969 ± 243	0.74 (0.18, 0.84)	12.46	0.45 (0, 0.75)	17.35
AMP_BZ_	2.18 ± 0.67	2.10 ± 0.51	2.13 ± 0.67	0.86 (0.66, 0.95)	5.53	0.75 (0.45, 0.90)	10.27
AUC_RF_	134 ± 60.1	125 ± 48.8	117 ± 40	0.88 (0.70, 0.95)	10.63	0.50 (0.04, 0.79)	16.64
AUC_BZ_	231 ± 83.3	219 ± 63.2	207 ± 55.8	0.91 (0.74, 0.97)	6.46	0.66 (0.27, 0.86)	11.97
1-min AUC	114 ± 34.0	112 ± 30.9	111 ± 32.2	0.94 (0.83, 0.98)	4.83	0.77 (0.47, 0.91)	9.70
AUC index	3.15 ± 0.53	3.06 ± 0.54	3.04 ± 0.39	0.84 (0.62, 0.94)	4.82	0.33 (-0.18, 0.69)	9.35
ORC_5_	0.37 ± 0.10	0.36 ± 0.10	0.35 ± 0.10	0.89 (0.72, 0.96)	6.95	0.73 (0.4, 0.89)	9.52
ORC_10_	0.21 ± 0.05	0.20 ± 0.04	0.20 ± 0.06	0.83 (0.58, 0.93)	6.33	0.71 (0.38, 0.89)	8.39
ORC_15_	0.15 ± 0.04	0.14 ± 0.03	0.15 ± 0.04	0.85 (0.64, 0.94)	5.13	0.74 (0.43, 0.90)	8.65
ORC_20_	0.11 ± 0.03	0.11 ± 0.03	0.11 ± 0.04	0.87 (0.69, 0.95)	4.83	0.69 (0.34, 0.88)	10.70
ORC_MF_	0.12 ± 0.05	0.12 ± 0.04	0.12 ± 0.05	0.74 (0.41, 0.90)	15.27	0.22 (-0.31, 0.63)	22.90

Statistics are shown as mean ± SD, ICC, 95% limits of agreement, and coefficient of variation for the three PORH responses (V_A_, V_B1_, V_B2_). Indices reported are biological zero (BZ), max flow as a percentage of biological zero (MF%BZ), amplitude form max flow normalized to biological zero (AMP_BZ_), functions of 4-min area under hyperemic curve expressed from resting flow and biological zero (AUC_RF_, AUC_BZ_) 1-min area under hyperemic response (1-min AUC) expressed from zero perfusion, ratio of 1-min AUC to 1-min of RF area under curve prior to occlusion (AUC index), overshoot rate of change from biological zero at 5s, 10s, 15s,20s (ORC_5s,10s,15s,20s_) and at time to max flux (ORC_MF_) after cuff deflation. Where applicable, values are represented as cutaneous vascular conductance (CVC).

## 4. Discussion

This is the first study to examine the reproducibility of kinetic indices (within the first minute) of the PORH response as well as multiple modes of PORH normalization (with respect to reference levels) utilizing LSCI. The main findings of this study are as follows: (i) among the conventional parameters assessed in this study, RF, MF, AMP_RF_, and 4-min AUC normalized to zero flux had good inter-day reproducibility (CV < 10%) while TMF and MF%RF were less reproducible and reliable; (ii) among the novel parameters introduced in this study, temporal variables based upon the first minute of hyperemia (i.e. 1-min AUC and AUC index) and those determining the kinetics of the hyperemic response in a stepwise fashion (i.e. ORC_5s,10s,15s,20s_) are reproducible (CV < 11%). Calculating the velocity of the hyperemic response using TMF (i.e. ORC_MF_) is an unreliable method (ICC = 0.22). Inter-day measurement of BZ (ICC = 0.64) is less reliable than RF (ICC = 0.89). However, BZ is a more reliable reference level for normalizing AMP (inter-day ICC AMP_BZ_ = 0.75 vs AMP_RF_ = 0.66) and AUC (inter-day ICC AUC_BZ_ = 0.66 vs AMP_RF_ = 0.50) with a reproducibility similar to the zero-flux normalization.

The PORH response has been typically normalized with respect to resting flow (RF) [16,17,19], biological zero (BZ) [26–28], or zero perfusion [20,22] (i.e. raw expression of flux). At least 3 min of occlusion is recommended to obtain a true LDF BZ value [29]. Normalizing the PORH response to BZ prior to data analysis removes any additive or confounding effects associated with non-hemodynamic changes (i.e. hemoglobin or interstitial electrolyte concentration) [29]. It has been shown that this correction does not alter the correlation between raw LDF and LSCI perfusion values but shifts the LSCI regression line towards origin [28]. We found that normalizing AUC as a function of BZ improves inter-day reproducibility and agreement compared to when normalized to RF. Thus, subtracting BZ is recommended when using absolute perfusion values. However, expressing LSCI data as a percentage change from the adjusted RF (i.e. resting flow subtracted from BZ) is not recommended because this correction introduces variability in adjusted RF that could impact normalization particularly at high flow measurements [28].

Previous investigators using LDF have defined BZ as the acquired flux signal in the absence of vascular flow and primarily the result of Brownian motion of macromolecules in the interstitial space [30]. However, the mechanisms explaining BZ may differ depending on the optical instrument used [30]. It has been reported that the relationship between LSCI and LDF at low flux values becomes non-linear and that LSCI perfusion tends to be higher than LDF for BZ [17]. This may partly be attributed to the pronounced non-linear dependence of LSCI speckle patterns on particle velocity [31]. Thus, the differences at low flux values (e.g. BZ) obtained from LSCI and LDF are suggestive of different probing mechanisms of the two techniques [32]. Using LSCI, we demonstrated that the acquired BZ signal does indeed vary (0.22 ± 0.08 AU/mmHg, inter-day CV = ~14%). This suggests the presence of velocity changing biological particles across the region of interest (i.e., the ventral forearm) during brachial artery occlusion. It should be noted that BZ has a higher variability than RF (inter-day CV = ~7%) and constitutes approximately 36% of the forearm cutaneous RF. One possible explanation for the higher variability in the BZ signal can be increased vasomotion during occlusion. A study using correlation mapping optical coherence tomography on human forearm demonstrated higher frequency of neurogenic, endothelial NO-dependent, and endothelial NO-independent effectors on cutaneous microvascular vasomotion during ischemia than resting flow [33]. Moreover, the inherent variability associated with the thermal-dependent stochastic Brownian motion may be more pronounced at lower vascular flow such as BZ.

MF (i.e. peak flow normalized to zero flux), a measure for the highest hyperemic flow and lowest vascular resistance was similarly acknowledged in other studies to be highly reproducible using LDF [17,22] and LSCI techniques [17,34]. The MF response can be expressed as a function of RF (i.e. AMP_RF_ and MF%RF) or BZ (i.e. AMP_BZ_ or MF%BZ). We observed MF%RF or MF%BZ was less reproducible compared to AMP_RF_ or AMP_BZ_ likely due to the wandering behavior of the RF or BZ. And, AMP_BZ_ is more reliable than the AMP_RF_ likely due to the lower absolute values of BZ. This highlights the impact of calculation method on data normalization and analysis. Studies using LDF or LSCI on diabetic patients and patients with coronary artery disease have demonstrated significantly reduced MF [35–37] or AMP_RF_ [37] compared to a healthy control group. A study using LDF showed that patients with primary Raynaud’s phenomenon expressed similar AMP_RF_ to the control group but had significantly lower MF response when normalized to zero perfusion [38]. This suggests that relying on one normalization technique to preserve reproducibility might mask the microhemodynamic cues imbedded in the other reference points of the PORH response relevant to a clinical or research setting.

Common PORH indices (i.e., MF and 4-min AUC) and PORH kinetic indices (i.e., TMF, 1-min AUC, ORC) may not reflect on identical underlying physiological mechanisms. TMF (i.e. time-to-peak), based upon the peak flux value, is dependent on a relatively proportionate amalgamation of myogenic, axonal reflex, NO-dependent, and NO-independent (e.g. EDHF) responses. While not directly addressed, kinetic responses associated with a relative shorter static time point (e.g. ORC_5s_, 1-min AUC) are suggested to incorporate a disproportionately greater contribution from myogenic and neuronal reflex than the delayed contribution of NO-independent factors like EDHF’s which is reflected in a relatively longer static time point (e.g. ORC_20s_, 4-min AUC) [33]. Given that microvascular dysfunction precedes vasculature morphological changes, assessing various shorter and longer temporal domains may be beneficial in eliciting the extent of neurogenic, NO-dependent, and NO-independent microhemodynamic impairments.

TMF is a dynamic measure of the kinetics of the PORH response and vascular resistance [39,40]. Results on this temporal index have been predominantly found using the LDF (CV~ 25%) [37] while the reproducibility of the index has not been previously established for LSCI. Clinically, TMF in type 2 diabetics is considerably shorter which is reflective of increased vascular resistance and impaired microvascular function [13]. However, TMF was not different between type 1 diabetics and healthy subjects [21]. One study suggests that a longer TMF (> 10s) is a diagnostic index for coronary artery disease [41]. Although, TMF may be a useful functional indicator, the knowledge on the duration corresponding to endothelial dysfunction is unclear. Our finding (mean TMF = 19 ± 5s) using LSCI as well others using LDF report TMF averages longer than 10s in healthy subjects [27,40,42,43]. Ultimately, the LSCI’s moderate inter-day reproducibility (CV ≈ 18%) of the TMF may be contributing to the inconsistent results on TMF which can lead to misrepresentation of the PORH response.

Overshoot rate of change (ORC) by definition is the restoration of blood flow at the post-ischemic extremity by quantifying the rate of perfusion change following cuff deflation, thereby providing an index of vascular resistance [22]. TMF has been typically used to study vascular resistance. However, the traditional approach of expressing TMF as a function of the flux (i.e. finding time using flux) is not reproducible and thus should not be incorporated in the calculation for ORC (15% < CV < 23%). Instead, a more reproducible alternative (5% < CV < 11%) occurs with fixed time points (i.e., finding flux using time). This alternative approach subdivides the hyperemic overshoot to define the changing hyperemic velocity in a reliable stepwise manner. Piecing the PORH response into fixed 5s intervals, ranging from 5-20s post-cuff deflation, nullified the temporal variations associated with time as a function of flux and rendered a reproducible stepwise quantification of the microhemodynamics. In addition to temporal considerations, the choice of normalization method in the calculation of ORC has not been standardized. Previous studies have normalized ORC to zero perfusion [35] and resting flow [21,27]. In the current study, ORC was normalized to BZ to assess the kinetics of PORH following occlusion. This method of normalization, using the mean flux at occlusion, does not extrapolate to RF or zero perfusion and evaluates the vascular function from the point of cuff deflation. Increased vascular resistance decreases the hyperemic flux signal. As a result, we suspect that the ORC values will be lower in patients with vascular pathology. In fact, one study suggests a significant reduction of the ORC_4s_ index in older-aged (> 65 years old) than younger individuals (< 45 years old) [44]. However, more studies are needed on this novel rate parameter to determine its clinical significance.

AUC is a temporal-paired index used to define the blood restoration volume due to ischemia. However, the hyperemic volume exceeds any flow debt incurred during occlusion [45]. There is no standardized duration for occlusion. Increasing the duration of occlusion increases the AUC and the MF [37,46]. Additionally, analysis methods of AUC are typically limited to one defined time point (e.g., 4-min AUC). However, assessing only one specific duration may mask the potential information obtained from the PORH response. In type 2 diabetics, reactive hyperemia induced by a 5-min brachial artery occlusion showed significantly diminished 1-min AUC response while no difference was detected in the 3- or 5-min AUC [13]. Thus, increased vascular resistance or microvascular dysfunction may have a greater effect earlier in the hyperemic response. Similarly, the AUC index, the ratio of 1-min AUC post-cuff deflation relative to 1-min AUC pre-cuff inflation, is a strongly associated index with diabetic microangiopathy compared to other PORH metrics like MF [13]. In light of its physiological importance however, our results showed poor data agreement (ICC = 0.33) for the inter-day assessment of the AUC index. This is likely due to the lower inter-day data agreement in the AUC of the resting flux. Furthermore, normalizing the AUC to RF (AUC_RF_) reduces the reproducibility (CV ~ 17%) of the response compared to when AUC is expressed from BZ (AUC_BZ_, CV ~ 12%) or zero flux (4-min AUC, CV ~ 9%).

### Limitations

There are methodological limitations to consider when applying this protocol. LSCI is a high-frame rate imaging technique that provides full-field coverage of the region of interest. However, LSCI is better suited for showing the relative changes in flow as it quantifies flux in arbitrary perfusion units instead of volume flow rate (ml/min) and has less signal penetration depth than LDF [17,47]. Furthermore, it is still unclear whether the absolute LSCI measurement is perfusion or velocity of the moving scatters [17]. And LSCI sensitivity to movement artifacts introduces bias in *post-hoc* perfusion analysis [39,48]. LSCI sensitivity to movement is also why static contraction or resistance exercise was not used as an ancillary measure to examine reactive hyperemia responses. Moreover, we were unable to balance for the sex ratio and provide reproducibility measures corresponding to sex. Lastly, the skin-model for studying the PORH response is sensitive to ambient temperature change. Specifically, it has been demonstrated that in LDF measurements BZ increases linearly and RF exponentially with increasing temperatures [29]. Therefore, controlling for temperature is important when studying PORH on the skin microvasculature [22,29,39].

## Conclusion

In summary, we demonstrated that biological zero is a reliable reference point for expressing the PORH response. Subtracting the BZ signal may remove the additive effects to flux while preserving the reproducibility value of the PORH response and therefore is recommended prior to data analysis. In addition, we showed that inter-day PORH kinetic indices such as ORC_5s,10s,15s,20s_ are more reliable for expressing the vascular resistance than TMF. TMF was shown to be an unreliable index for calculating the velocity of the PORH response. Collectively, using less reliable indices or normalization methods incorporates random error in the data thereby lowering the statistical power and potentially misrepresenting the hyperemic response. In this study we have suggested data collection and analysis methods to improve the longitudinal assessment of the PORH signal using LSCI. This may facilitate the translation of PORH to a clinical setting and its application as a surrogate marker for the detection of microvascular dysfunction.

## Supporting information

S1 Data LSCI data used for statistical analysis. (XLSX)

S2 Data ORC data used for statistical analysis. Includes the analysis of the normal distribution of TMF data.
(XLSX)
